# Anemia and its predictors among chronic kidney disease patients in Sub-Saharan African countries: A systematic review and meta-analysis

**DOI:** 10.1371/journal.pone.0280817

**Published:** 2023-02-02

**Authors:** Mitku Mammo Taderegew, Alemayehu Wondie, Tamene Fetene Terefe, Tadesse Tsehay Tarekegn, Fisha Alebel GebreEyesus, Shegaw Tesfa Mengist, Baye Tsegaye Amlak, Mamo Solomon Emeria, Abebe Timerga, Betregiorgis Zegeye

**Affiliations:** 1 Department of Biomedical Sciences, College of Medicine and Health Sciences, Wolkite University, Wolkite, Ethiopia; 2 Department of Nursing, College of Medicine and Health Sciences, Wolkite University, Wolkite, Ethiopia; 3 HaSET Maternal and Child Health Research Program, Shewarobit Field Office, Shewarobit, Ethiopia; University of Cape Town Faculty of Science, SOUTH AFRICA

## Abstract

**Introduction:**

Anemia is a serious complication of chronic kidney disease (CKD) with a significant adverse outcome on the burden and progression of the disease. Hence, the study intended to assess the pooled prevalence of anemia and its predictors among CKD patients in Sub-Saharan African nations.

**Methods:**

To identify the relevant studies systematic searches were carried out in Medline, EMBASE, HINARI, Google Scholar, Science Direct, and Cochrane Library. From selected studies, data were taken out with a standardized data extraction format prepared in Microsoft Excel. Inverse variance (I^2^) tests were employed to evaluate the heterogeneity across the included studies. Due to substantial heterogeneity among the studies, a random-effects meta-analysis technique was employed to estimate the pooled prevalence of anemia. Subgroup analysis, sensitivity analysis, and meta-regression analysis were carried out to search the possible bases of heterogeneity. Funnel plot symmetry, Begg’s test, and Egger’s regression test were employed to assess the existence of publication bias. In addition, factors associated with anemia among CKD patients were examined. All statistical analyses were carried out with STATA™ Version 14 software.

**Results:**

A total of 25 studies with 5042 study participants were considered in this study. The pooled prevalence of anemia among CKD patients was estimated to be 59.15% (95% CI, 50.02–68.27) with a substantial level of heterogeneity as evidenced by I^2^ statistics (I2 = 98.1%; p < 0.001). Stage of CKD (3–5) (pooled odds ratio (POR) = 5.33, 95% CI:4.20–6.76), presence of diabetes mellitus (POR = 1.75, 95% CI: 1.10–2.78), hemodialysis history (POR = 3.06, 95% CI: 1.63–5.73), and female sex (POR = 2.50, 95% CI: 1.76–3.55) were significantly related with anemia.

**Conclusions:**

More than half of CKD patients were suffering from anemia. Stage of CKD, presence of DM, hemodialysis history, and being female sex were factors associated with anemia among CKD patients.

## Introduction

Chronic kidney disease (CKD) is an emerging global public health issue that places a significant financial burden on both the patients’ families and the healthcare delivery system. CKD is a major basis of morbidity and mortality in both developed and developing nations, affecting more than 10% of the worldwide population in 2015 [1].

Evidences have shown that African descendants had a higher risk of developing CKD and advancement to end-stage renal disease (ESRD) [2, 3]. Due to rapid urbanization, adoption of Western lifestyles, increasing prevalence of obesity and physical inactivity, rapid population growth, and the growing prevalence of CKD risk factors with disproportionate impact in the developing nations, the rate of CKD is increasing promptly in the Sub-Saharan African nations with an estimated prevalence of 15.8% in 2018 [2–4].

Anemia is a communal and serious complication of CKD with a significant adverse outcome on the burden and progression of the disease. It is a common and avoidable risk factor for many adverse outcomes in CKD patients and it is related with a reduced quality of life, as well as increased morbidity and mortality [5, 6]. The presence of anemia among CKD patients is a significant indicator of cardiovascular events and has therefore been largely associated with a greater likelihood of hospitalization and prolonged hospital stay, poor quality of life, as well as increased morbidity and mortality [7]. According to the Kidney Disease Improving Global Outcomes (KDIGO) Anemia Work Group, anemia in CKD exists when the hemoglobin (Hb) value is <13 g/dL for men and <12 g/dL for women [8]. The documented causes of anemia in CKD patients are multifactorial and include deficiency of erythropoietin, resistance to erythropoietin, reduced red blood cell lifespan, iron deficiency, chronic inflammatory process, and uremic milieu [6, 9, 10].

Since anemia is a common finding among CKD patients and is related with high mortality, early screening and optimal intervention have been shown to reduce morbidity and mortality and improve the quality of life of the patients [6, 7, 11–13]. The burden of anemia among CKD patients varies greatly across many regions. For instance, it has been shown that 91.8% of CKD patients in central South Africa [14], 14.0% in Nigeria [15], and 39.5%-89.5% in Ethiopia [16, 17] suffered from anemia. Even though various primary studies in Sub-Saharan African nations revealed the prevalence of anemia among CKD patients, the majority of these studies was single-centered and had small sample sizes. The results of these studies also showed significant variation and inconsistency regarding the prevalence of anemia in the region. These uncertainties and inconsistency of findings across regions make it difficult for policymakers to make decisions based on such studies.

It is therefore imperative to estimate the overall magnitude of anemia and its associated factors among patients presenting with CKD. Hence, this study intended to estimate the overall prevalence of anemia and its associated factors among patients presenting with CKD in Sub-Saharan African nations. The results of the study would serve as baseline data for policymakers and other stakeholders to plan and apply proper interventions that stress routine screening, and appropriate management of anemia among patients presenting with CKD. The results will also be helpful for other investigators, as baseline data for further investigation. It is also hoped that the findings will help clinicians to appreciate the magnitude of anemia and implement appropriate interventions among CKD patients in the clinical setup.

## Materials and methods

A systematic review and meta-analysis were carried out to estimate the pooled prevalence of anemia in CKD patients. The review was undertaken according to the guideline of the Preferred Reporting Items for Systematic reviews and Meta-Analysis (PRISMA) checklist (S1 Table) [18]. All literature accessible until January 2022 was considered in the study.

### Searching strategy and study selection

To ascertain all eligible literature that reports the prevalence of anemia and its predictors among patients presenting with CKD in sub-Saharan African nations, a systematic exploration of the literature was conducted by three authors (MMT, AW, TFT) through Medline (PubMed), EMBASE, HINARI, Google Scholar, Science Direct, CINAHL, Popline, Cochrane Library African Journals Online (AJOL), and grey literature. Additionally, the reference lists of each retrieved article were also searched manually for search optimization. The search was carried out using the following key search terms and phrases: “anemia”, “anaemia”, “associated factors”, “risk factors”, “chronic renal impairment”, “chronic kidney injury”, “chronic renal insufficiency”, “chronic kidney disease”, “Sub Saharan Africa”, and “names of each the sub-Saharan Africa countries”. Boolean operators like “AND” and “OR” were used to combine search terms. The search of the article was limited to full texts, free articles, and published in peer-reviewed journals in the English language. Then, based on the eligibility criteria, two authors (TTT & FAG) independently reviewed all potential article titles, abstracts, and full-text quality. Finally, the screened articles were compiled together.

#### Inclusion and exclusion criteria

All studies in sub-Saharan African countries that reported the prevalence of anemia and its predictors among CKD patients were illegible for the study. However, the studies that did not report our outcome of interest (prevalence anemia and/ or its predictors) or if it is not possible to calculate from the available data; studies which were not fully accessible even after e-mailing the primary author twice; and studies having a low-quality score in accordance with the given quality assessment criteria were excluded from this study.

### Data extraction and quality assessment

The selected articles were thoroughly and independently examined by the other two authors (STM & BTA), and the necessary data for the review were collected and summarized using a straightforward data extraction spreadsheet format prepared in Microsoft Office Excel software. If inconsistencies among data extractors were detected, a third author acted as the final arbiter. In instances of incomplete data, emails were sent to the corresponding author twice in an attempt to get further information, or calculations were conducted using the available information.

The data extraction tool comprises the name of the first author, publication year, country where the study was conducted, design of the study, sample size, the prevalence of anemia with 95% confidence interval (CI), dialysis status, and each specific factor (factors if two or more studies considered them as predictors of anemia among CKD patients). For every associated factor, in order to calculate the odds ratio, the data from the primary studies were extracted by three authors (MSE, AT, MMT) in the form of two by two tables.

The methodological quality of each incorporated study was evaluated by two authors (AW, TFT) independently, using the Newcastle-Ottawa scale (NOS) quality valuation tool that was adopted for the quality evaluation of cross-sectional studies [19]. The tool comprises three major parts; the first part is rated up to five stars, and measures the methodological quality of each study. The second part of the instrument assesses the comparability of each study and gives two points. The third part measures the quality of the original articles with regard to the appropriateness of the statistical methods employed to analyze the data and can be rated out of three stars. Any disagreement between the reviewers regarding each article was resolved by all authors, forwarding their suggestions and the final decision was reached by consensus. Finally, the studies were taken into the analysis if they scored ≥5 out of 10 points in three domains of ten modified NOS constituents for observational studies (S2 Table).

Additionally, the risk of bias in selected studies was evaluated using the Hoy et al. [20] risk of bias tool for prevalence studies. The tool has ten items, each of which was given a score of 1 (yes) or 0 (no). The scores from all the items were added up to generate an overall quality score, which ranged from 0 to 10. Two authors (TT, FAG) independently conducted the risk of bias assessment of the included articles. When the available data were not sufficient to help make a decision for a certain item, we contacted the corresponding authors for additional information and if uncertainty persisted we decided to grade that item as 0 (failure to satisfy a specific item) meaning a high-risk of bias. Then each study was ranked as being of low, moderate, or high methodological quality according to the number of items judged as “yes (low risk of bias)”. Studies were considered to be of high-, moderate- and low quality based on scores that were greater than 8, between 6 and 8, and equal to or lower than 5, respectively (S3 Table).

### Statistical methods and analysis

For statistical analysis, the extracted data were imported into STATA version 14 software (StataCorp LP, College Station, TX, USA). The heterogeneity across the recorded studies was evaluated by using the heterogeneity I^2^ test and its p-values, with I^2^ values of below 25, 25–75, and above 75% demonstrating low, medium and, high heterogeneity, respectively [21]. The tests conducted for this meta-analysis show that there is significant high heterogeneity among the included studies (I^2^ = 98.1%, P-value 0.001).

As a result, a random-effects meta-analysis model was applied to estimate the pooled prevalence of anemia among CKD patients. Using a forest plot, the pooled prevalence with a 95% CI was produced and displayed.

Furthermore, subgroup analysis and meta-regression were employed to detect the probable cause of heterogeneity. In addition, sensitivity analysis was conducted to evaluate the relative effect of each study on the overall estimate by omitting each study one by one. Besides, the possibility of publication bias was also evaluated by using funnel plot symmetry, Egger’s regression test, and Begg’s test. Finally, the different factors associated with anemia among CKD patients were presented using pooled odds ratios (PORs) with a corresponding 95% CI.

## Results

### Selection of the studies

**Fig 1:**
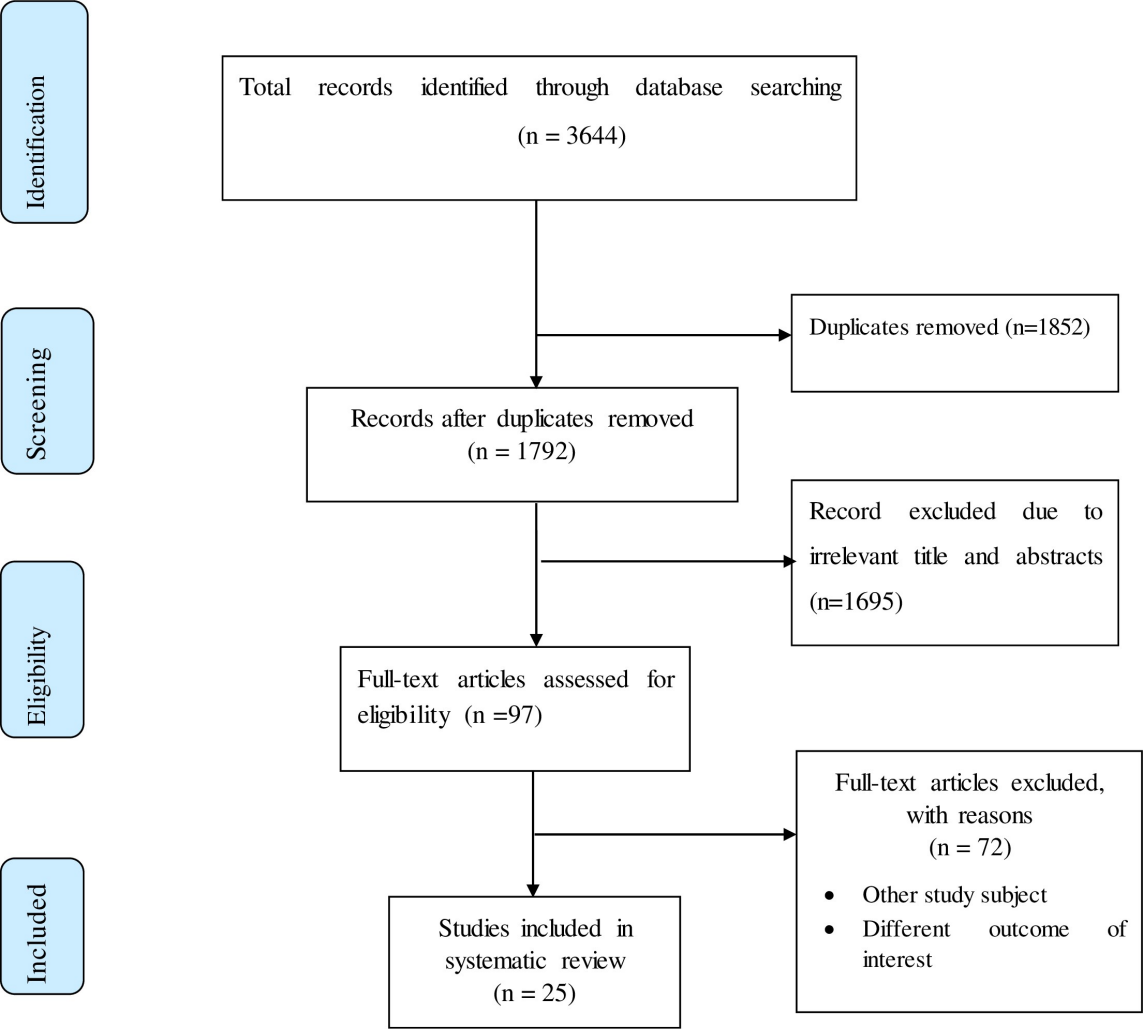
Flow chart showing the selection of studies for the systematic review and meta-analysis of prevalence and predictors of anemia among CKD patients in Sub-Saharan African countries.

A total of 3644 records concerning the prevalence and predictors of anemia among CKD patients in sub-Saharan African regions were collected from the databases of PubMed, HINARI, EMBASE, Science Direct, Google Scholar, Cochrane Library, and grey literature. Of these studies, 1852 articles were removed due to repetition. After assessing the title and abstract, 1695 articles were removed from the remaining 1792 studies as they were found to be non-applicable for this systematic review and meta-analysis. The remaining 97 studies were then assessed for eligibility based on the pre-defined criteria, which resulted in the further exclusion of 72 studies. Finally, 25 studies that satisfied the eligibility criteria were considered in the analysis (Fig 1).

### Baseline characteristics of included studies

A total of 25 original articles that revealed the prevalence and predictors of anemia among 5042 CKD patients in sub-Saharan African regions which were published between 2010 and 2021 were incorporated in this systematic review and meta-analysis. These studies were carried out in South Africa (5 studies) [14, 22–25], Nigeria (6 studies) [15, 26–30], Ethiopia (5 studies) [16, 17, 31–33], Tanzania (2 studies) [34, 35], Kenya (2 studies) [36, 37], Cameron (3 studies) [38–40], Sudan (1 study) [41], and Uganda (1 study) [42]. Of the studies incorporated in the final analysis, 22 (88.0%) of them were cross-sectional, 2 (8.0%) were case-control, and 1 (4.0%) was a prospective cohort study. The sample size of the incorporated studies ranged from 49 in South Africa [14] to 792 in Tanzania [34] (Table 1).

**Table 1:** Characteristics of included studies in the systematic review and meta-analysis of anemia among CKD patients, in Sub-Saharan African countries, 2022.

Authors	Publication Year	Country	Sample Size	Anemia	Prevalence with 95% CI	Quality (NOS)/ 10 pts	Definition of anemia	Definition of CKD	Predictors
Adera et al. [31]	2019	Ethiopia	251	162	64.50 (58.58, 70.42)	7	WHO definition	Not specified	Residence, BMI, hemodialysis history
Nalado et al. [22]	2019	S. Africa	353	152	43.10 (37.93, 48.27)	7	WHO definition	Not specified	Stage of CKD, DM
Alemu et al. [32]	2021	Ethiopia	387	207	53.50 (48.53, 58.47)	8	WHO definition	eGFR<60 mL/min/ 1.73 m^2^ for ≥ 3 months	Sex, hemodialysis history, stage of CKD, DM, protein urea, Hypertension
Meremo AJ et al. [34]	2017	Tanzania	792	249	31.40 (28.17, 34.63)	7	WHO definition	Not specified	Sex, heart failure, stage of CKD
Akinola OL et al. [26]	2018	Nigeria	55	30	54.50 (41.34–67.66)	5	WHO definition	Not specified	Age, sex, DM, decline eGFR
Ljoma et al. [27]	2010	Nigeria	364	282	77.50 (73.21, 81.79)	6	Hb <12g/dL	eGFR<60 mL/min/ 1.73 m^2^ for ≥ 3 months or albuminuria	HIV, collagen vascular disease, chronic glomerulonephritis
Maina et al. [36]	2016	Kenya	212	142	67.00 (60.67,73.33)	6	WHO definition	Not specified	stage of CKD, DM, systemic lupus erythematous
Raji et al. [28]	2018	Nigeria	157	67	42.70 (34.96, 50.44)	8	K/DOQI.	eGFR<60 mL/min/ 1.73 m^2^ for ≥ 3 months or albuminuria	Sex, stage of CKD, hemodialysis history
Abate et al. [16]	2013	Ethiopia	57	51	89.50 (81.54, 97.46)	8	WHO definition	eGFR<60 mL/min/ 1.73 m^2^ or albuminuria	…….
George C et al. [23]	2018	South Africa	94	40	42.60 (32.60, 52.60)	7	K/DOQI	eGFR<60 mL/min/ 1.73 m^2^	……‥
Iyawe & Adejum [29]	2018	Nigeria	100	90	90.00 (84.12, 95.88)	5	WHO definition	eGFR<60 mL/min/ 1.73 m^2^ for ≥ 3 months or marked kidney damage	Stage of CKD
Emmanuel et al. [30]	2020	Nigeria	113	100	88.50 (82.62, 94.38)	7	WHO definition	eGFR<60 mL/min/ 1.73 m^2^ for ≥ 3 months or marked kidney damage	……
Valerian et al. [37]	2019	Kenya	118	57	48.30 (39.28, 57.32)	9	Hb <10g/dL	eGFR<60 mL/min/ 1.73 m^2^ for ≥ 3 months or marked kidney damage	…….
Bashir et al. [41]	2016	Sudan	70	51	72.90 (62.49, 83.31)	8	WHO definition	eGFR<60 mL/min/ 1.73 m^2^ or hemodialysis	…….
Fiseha T et al. [17]	2019	Ethiopia	177	70	39.50 (32.30, 46.70)	8	WHO definition	eGFR<60 mL/min/ 1.73 m^2^ or albuminuria	…….
Nalado AM et al. [24]	2018	South Africa	258	91	35.30 (29.47, 41.13)	6	K/DOQI	eGFR<60 mL/min/ 1.73 m^2^	……‥
Francois et al. [38]	2015	Cameroon	95	75	78.90 (70.70, 87.10)	7	K/DOQI	Not specified	…….
Halle et al. [39]	2013	Cameroon	113	93	82.70 (75.73, 89.67)	6	Hb <11 g/dL	Not specified	…………‥
Haupt, & weyers [14]	2016	South Africa	49	45	91.80 (87.9, 95.7)	5	K/DOQI	Not specified	…….
Namuyimb et al. [42]	2018	Uganda	93	14	37.80 (27.95, 47.65)	7	Hb < 11.5 g/dL	eGFR<60 mL/min/ 1.73 m^2^ or any eGFR with proteinuria	……
Nalado et al. [25]	2020	S.Africa	312	103	33.00 (27.78, 38.22)	8	WHO definition	eGFR<60 mL/min/ 1.73 m^2^	……
Iyawe IO et al. [15]	2018	Nigeria	100	14	14.00 (7.20, 20.80)	7	K/DOQI	eGFR<60 mL/min/ 1.73 m^2^ for ≥ 3 months	……
Ruggajo et al. [35]	2019	Tanzania	233	161	69.00 (63.06, 74.94)	8	WHO definition	Not specified	…‥
Kaze et al. [40]	2020	Cameroon	105	91	86.70 (80.20, 93.20)	6	K/DOQI	eGFR<60 mL/min/ 1.73 m^2^	…‥
Kidanewold et al. [33]	2021	Ethiopia	384	169	44.00 (39.00, 48.90)	8	WHO definition	eGFR<60 mL/min/ 1.73 m^2^	Cardiovascular disease, DM, stage of CKD

BMI: Body mass index; CI: Confidence interval; CKD: Chronic kidney disease; DM: Diabetes mellitus; eGFR: Estimated glomerular filtration rate; g/Dl: gram per deciliter; Hb: Hemoglobin; HIV: Human immune deficiency virus; K-DOQI: Kidney Disease Outcome Quality Initiative; NOS: Newcastle-Ottawa scale; WHO: World Health Organization

### Meta-analysis

#### Prevalence of anemia among CKD patients

**Fig 2:**
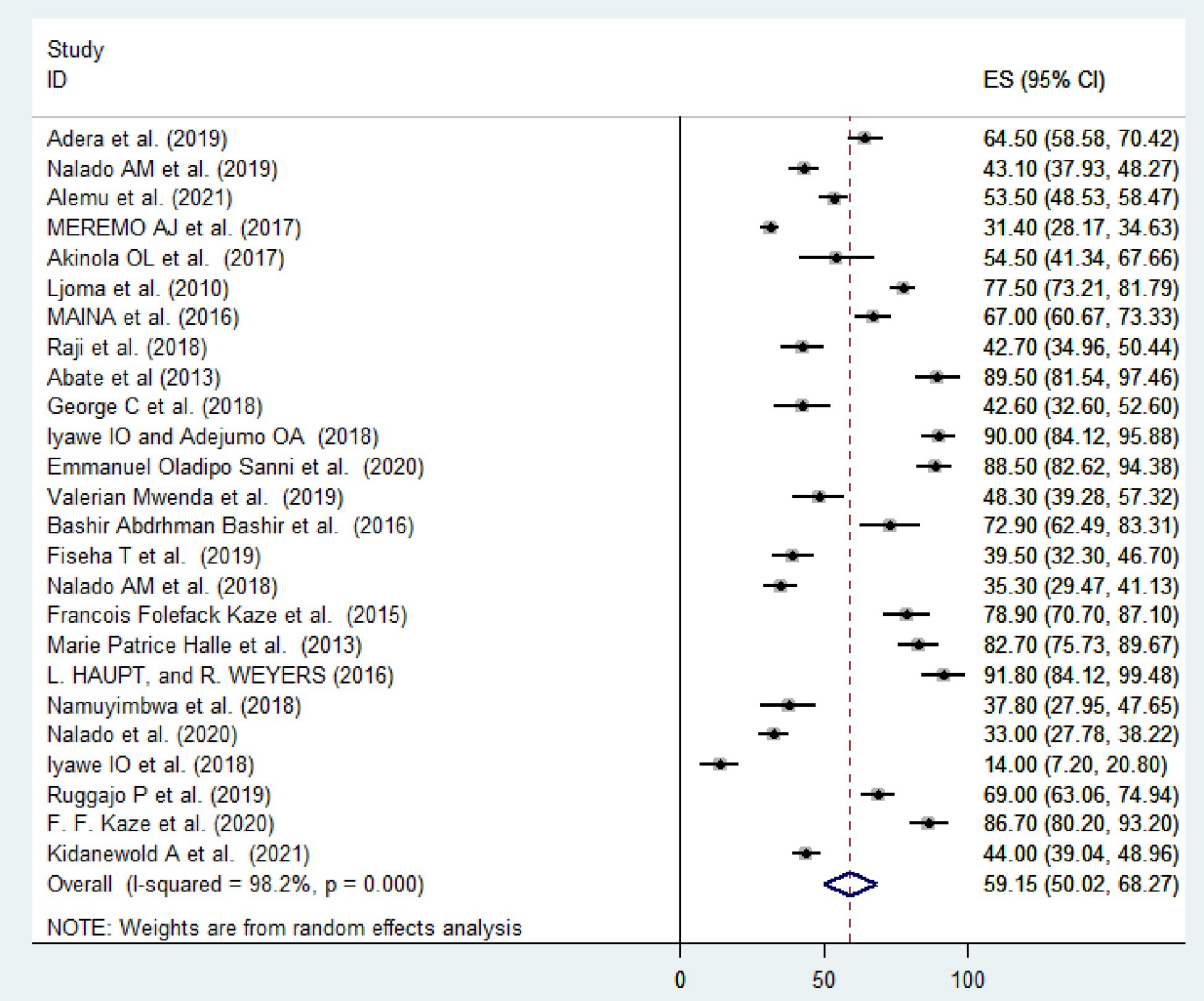
Forest plot of the pooled prevalence of anemia among CKD patients in Sub-Saharan African countries, 2022.

The pooled prevalence of anemia among CKD patients in Sub-Saharan countries was estimated to be 59.15% (95% CI, 50.02–68.27) with a substantial level of heterogeneity as evidenced by I^2^ statistic (I^2^ = 98.1%; p < 0.001), indicating a great variability in the prevalence of anemia among patients presented with CKD across the studies. Hence, a random effect model was employed to determine the pooled prevalence of anemia among CKD patients in sub-Saharan African regions (Fig 2).

#### Subgroup analysis

To pinpoint the source of heterogeneity, subgroup analyses based on the study area, year of publication, sample size, dialysis status, and bias risk of the included studies were conducted. Based on the subgroup analysis, the pooled prevalence of anemia in the included studies ranged between 37.8% in Uganda and 83.30% in Cameroon, 54.76% among studies published after the years of 2015, and 81.63% up to the year 2015, and 65.51% among studies with moderate risk of bias and 51.00% among study with low-risk of bias.

Another subgroup analysis based on the sample size showed the highest prevalence (67.68%) of anemia was detected among the studies with a sample size of less than 150; whereas the lowest (50.04%) was observed among the studies with a sample size of 150 and above. Furthermore, the pooled prevalence of anemia in studies among dialysis and pre-dialysis patients was 71.01 and 55.42%, respectively (Table 2). In addition to subgroup analysis, a meta-regression analysis for the included studies was also carried out, by taking into consideration of factors such as publication year and sample size although none of these variables was found to be statistically significant.

**Table 2:** Subgroup analysis of the prevalence of anemia among CKD patients in Sub-Saharan African countries, 2022.

Sub-group	Category	Number of studies	Sample size	Prevalence (95% CI)	Heterogeneity	P-value	I^2^ (%)	Tau-squared
Country	Ethiopia	5	1256	58.06 (43.45, 72.67)	119.09	0.000	96.6	267.40
	S. Africa	5	1066	49.07 (30.34, 67.79)	174.24	0.000	97.7	443.66
	Tanzania	2	1025	50.11 (13.27, 86.96)	118.8	0.000	99.2	700.93
	Nigeria	6	889	61.31 (37.6, 84.97)	406.44	0.000	98.8	858.83
	Kenya	2	330	57.94 (39.62, 76.25)	11.07	0.001	91.0	159.05
	Sudan	1	70	72.90 (62.49, 83.31)	0.00	…	…	0.000
	Cameroon	3	313	83.30 (78.99, 87.61)	2.19	0.335	8.5	1.24
	Uganda	1	93	37.80 (27.94, 47.65)	0.00	---	…	0.000
Publication years	<2015	21	4413	54.76 (45.13, 64.39)	1015.95	0.000	98.0	492.61
	≤2015	4	629	81.63 (76.43, 86.83)	7.32	0.062	59.0	16.27
Sample size	≥150	12	3880	50.04 (40.25, 59.83)	462.70	0.000	97.6	290.94
	<150	13	1162	67.68 (53.28, 82.08)	534.02	0.000	97.8	682.86
Risk of bias	Low risk	11	3040	51.00 (41.14, 60.87)	319.07	0.000	96.9	265.46
	Moderate	14	2002	65.51 (52.28, 78.75)	711.00	0.000	98.2	624.19
Dialysis status	Pre-dialysis	19	3690	55.42 (45.26, 65.59)	885.21	0.000	98.0	496.85
	Dialysis	6	1352	71.01 (47.48, 94.55)	418.38	0.000	98.8	850.62

#### Sensitivity analysis

**Fig 3:**
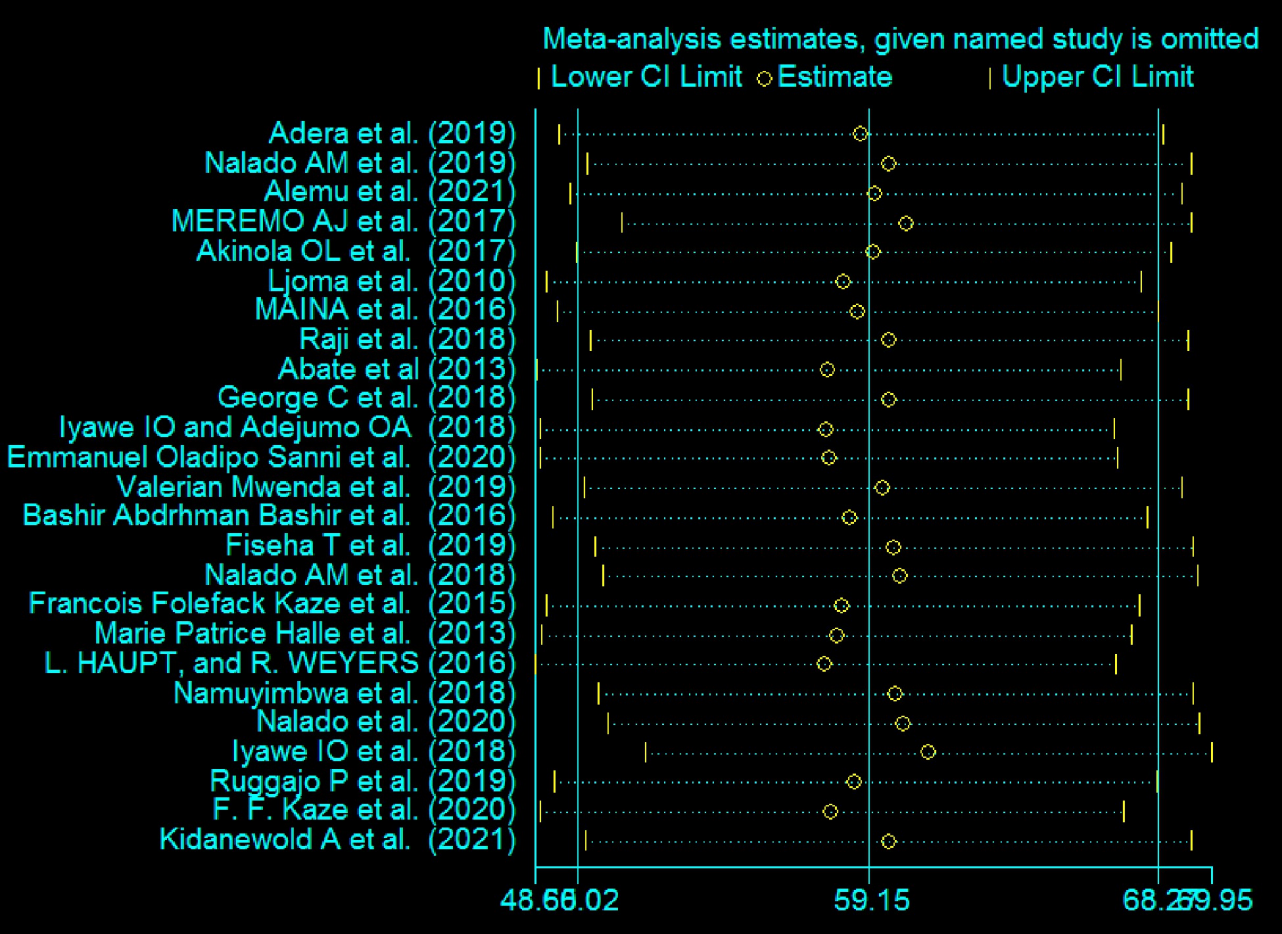
Results of sensitivity test of 25 studies.

A leave-one-out sensitivity analysis was also conducted to determine the cause of heterogeneity. The results of the random-effects model’s sensitivity analysis indicated that no single study had an impact on the overall prevalence of anemia among CKD patients (Fig 3).

#### Publication bias

**Fig 4:**
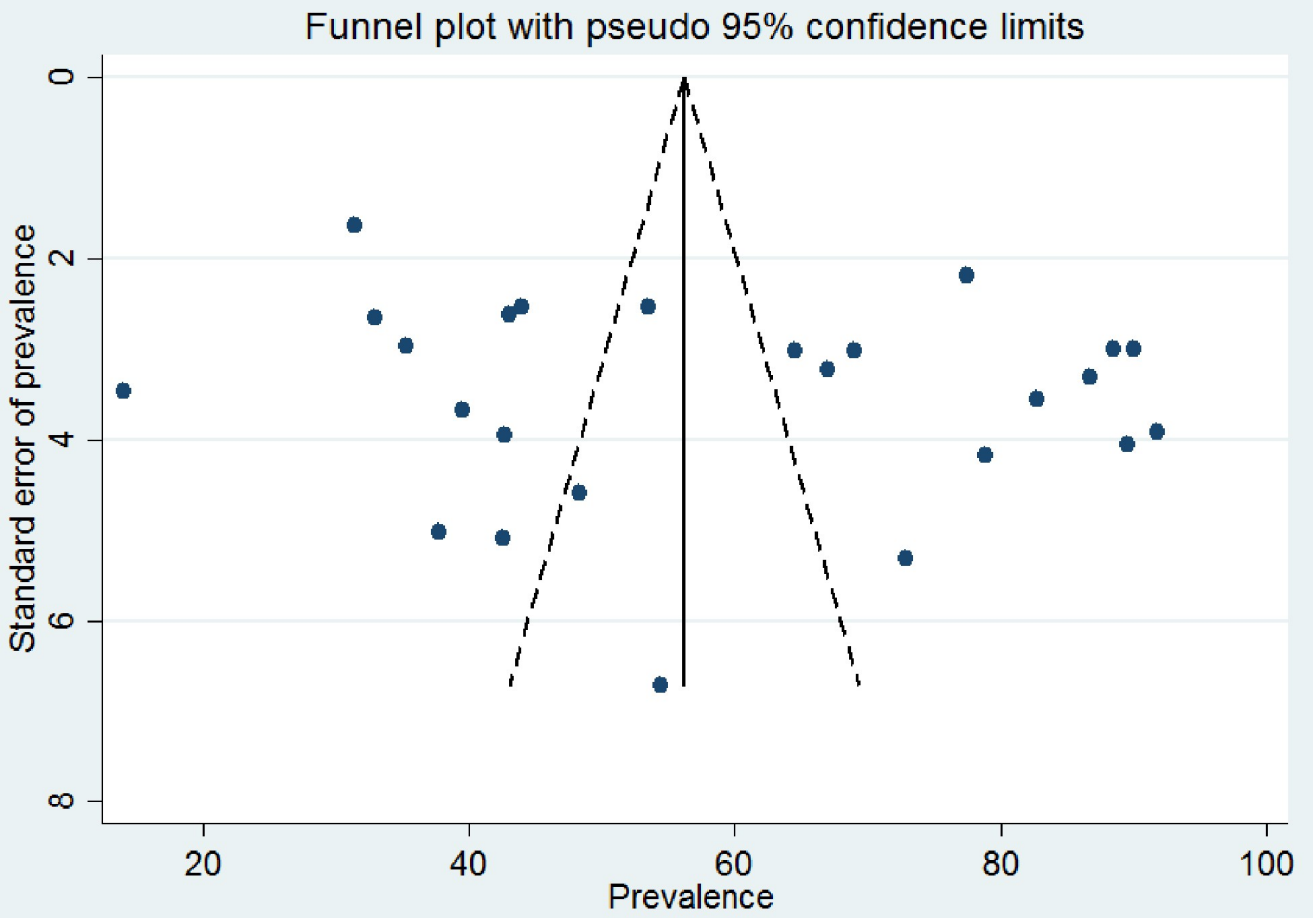
Test of publication bias of 25 studies using a funnel plot.

The publication bias was evaluated with a funnel plot, Beggs’ and Eggers’ tests. The funnel plot (Fig 4) was symmetric and Egger’s regression test (P = 0.607), as well as Begg’s test (P = 0.191), provided no evidence of publication bias.

#### Factors associated with anemia among CKD patients

In this systematic review and meta-analysis stage of CKD, the presence of diabetes mellitus (DM), female sex, and history of hemodialysis were recognized as factors significantly associated with the presence of anemia among CKD patients. For each identified factor except sex, sensitivity analysis was also carried out by excluding each study one by one, but the result showed that there was no strong evidence for the effect of a single study on the overall result.

**Fig 5:**
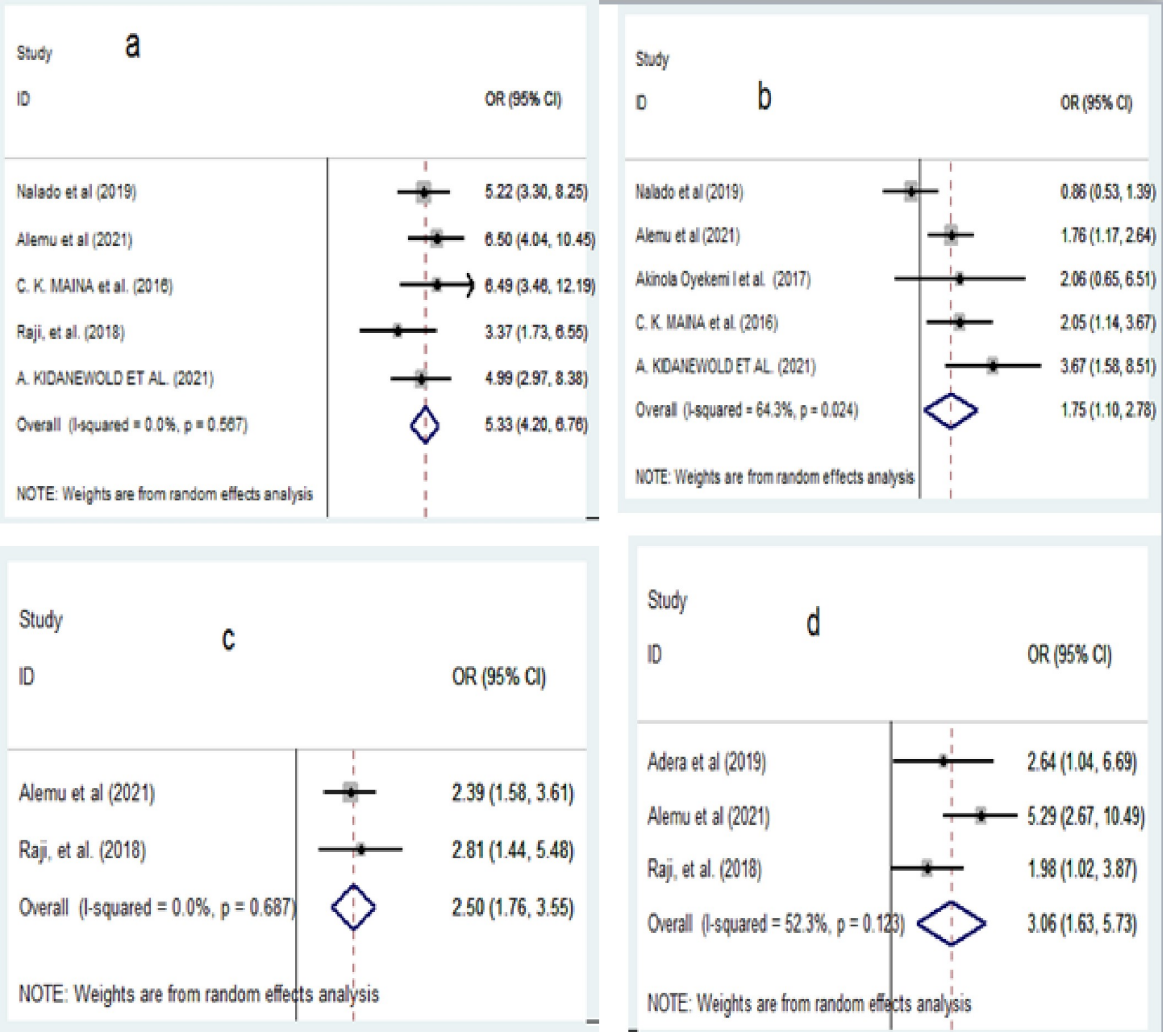
Forest plot showing pooled odds ratio of the factors associated with anemia among CKD patients in Sub-Saharan African countries. A: stage of CKD; b: diabetes mellitus; c: sex of the study participants; d: history of hemodialysis.

Five studies [22, 28, 32, 33, 36] evaluated the association between the stage of CKD and the presence of anemia among CKD patients. The pooled odds ratio showed that CKD patients with stage 4–5 were five times more likely to have anemia (POR = 5.33, 95% CI:4.20–6.76) than those with stage 1–3 (Fig 5A). Similarly, the analysis of 5 studies [22, 26, 32, 33, 36] showed that the presence of DM among CKD patients was significantly associated with anemia than those without DM (POR = 1.75, 95% CI: 1.10, 2.78) (Fig 5B). Furthermore, the pooled result from the two studies [28, 32] considered in the meta-analysis have shown that being female was two times more likely to have anemia than being male (POR = 2.50, 95% CI: 1.76–3.55) (Fig 5C). Moreover, the association between hemodialysis history and anemia was evaluated according to the findings from three studies [28, 31, 32]. The pooled odds ratio from these studies indicated that CKD patients with a history of hemodialysis were three times more likely to have anemia than patients without a history of hemodialysis (POR = 3.06, 95% CI: 1.63–5.73) (Fig 5D).

## Discussion

This systematic review and meta-analysis deliver evidence of an estimated pooled prevalence of anemia among CKD patients in Sub-Saharan African countries. Based on the review, the pooled prevalence of anemia was 59.15% (95% CI, 50.02–68.27). The finding is in line with the studies conducted in Turkey (55.9%) [43], Malaysia (60.3%) [44], Spain (58.5%) [45], Nepal (53.6%) [46] and China (51.5%) [47]. On the other hand, the prevalence of anemia among CKD patients is lower than the prevalence reported in Brazil (86.1%) [48], Malaysia (75.8%) [49] and Pakistan (80.5%) [50]. On the other hand, the observed magnitude of anemia in this finding was greater than the results in Korea (45.0%) [51], Nepal (47.8%) [52], Japan (32.3%) [53], and the USA (15.4%) [54].

These wide discrepancies in the burden of anemia among CKD patients might be explained by the differences in the socio-economic status, the definition of anemia, the study population, methodology, inclusion and exclusion criteria of the study, the stage of CKD, and the quality of healthcare services.

Sub-group analysis of this study indicated that the prevalence of anemia among dialysis patients 71.01% (95% CI: 47.48–94.55) was higher than their counterparts, 55.42% (95% CI: 45.26–65.59). It is not surprising to see anemia more commonly among dialysis patients than non-dialysis patients in this study, as the various studies revealed that erythropoietin deficiency and losing blood, either through blood tests or during dialysis were common among dialysis patients [28, 31, 32]. The prevalence of anemia also varies greatly across the regions with the highest prevalence observed in Cameroon 83.30% (95% CI: 78.99–87.61), followed by Sudan 72.90% (95% CI: 62.49–83.31) while the lowest prevalence was seen in Uganda 37.80% (95% CI: 27.94,-47.65). Since the study participants in Cameroon and Sudan were patients on dialysis, observing the high prevalence of anemia in these countries may support the acceptability of the above explanation. Moreover, these discrepancies in the prevalence of anemia among CKD patients across the regions may be due to the differences in methodology that the study employed and in the clinical features of the study participants, particularly the stage of CKD.

Predictors of anemia were also assessed in the present systematic review and meta-analysis. History of hemodialysis was identified as a predictor of anemia among CKD patients in which CKD patients with a history of hemodialysis were three times more likely to be suffered from anemia than those patients without a history of hemodialysis. This is most likely because hemodialysis-requiring patients were those with a long duration of the disease, advanced renal disease, and comorbidities, in which the presence and severity of anemia were prevalent [31, 32].

In this finding, the sex of the study participants was significantly associated with the presence of anemia in which being female is stated to be a non-modifiable risk factor for the occurrence of anemia among CKD patients, yielding a similar result to a study carried out in Turkey [43], Malaysia [44], Italy [55], United States [56], and Iran [57]. This may be simply elucidated by the biological susceptibility of the inevitable blood loss at the time of menstruation and pregnancy in pre-menopausal women as well as dietary inadequacy [28, 43].

The study also showed that the stage of CKD was significantly associated with the occurrence of anemia among CKD patients. It was found that the risk of anemia was gradually increased as the CKD advanced, which corresponds well with the results of earlier studies conducted in South Korea [51], Malaysia [44], Pakistan [50], USA [56], Greece [58], and Singapore [59]. Increased risk of anemia in advanced renal disease patients could be due to the known pathogenesis of CKD mainly impaired production of erythropoietin as kidney function worsens, urinary erythropoietin losses, metabolic disturbances, and reduced red blood cells life span because of uremic environment and the possible effect of circulating uremic-induced inhibition of erythropoiesis [9, 22, 36].

Finally, CKD patients with DM had an approximately two-fold increased risk of anemia than those without DM. Likewise; an increase in the risk of anemia among CKD patients with DM has been reported by various studies carried out in the United States [56], Greece [58], and Nepal [60]. This is mainly due to the impact of diabetes-related chronic hyperglycemia. Chronic hyperglycemia can cause a persistent hypoxic environment in the renal interstitium which results in impaired erythropoietin production. Additionally, erythrocyte precursor cells in the bone marrow may be exposed to prolonged direct glucose toxic effects, or mature red blood cells may be damaged by oxidative stress, both of which can influence erythrocyte production and red cell survival in patients with prolonged hyperglycemia. Chronic inflammatory activity, elevated levels of advanced glycation end products (AGEs), hyporesponsiveness to erythropoietin, the effects of oxidative stress, and anti-diabetic medicines are also other possible causes of anemia in DM patients [22, 26, 58].

### Limitations of the study

There are a few potential significant limitations to this systematic review and meta-analysis. First of all, there is no uniformity of CKD definitions and anemia cut-off points in the included studies that may affect the estimation of the combined prevalence. Second, it might be challenging to apply the conclusions from a small number of sub-Saharan African nations included in this meta-analysis to all CKD patients in that region. Third, most of the studies incorporated in the review were cross-sectional in nature. As a consequence, a cause-effect relationship cannot be established. Hence further study with a strong study design and by considering other causes of anemia should be conducted. Additionally, there has been a substantial heterogeneity among the included studies and the cause/s for the heterogeneity thus remains elusive, which could make it difficult to interpret the findings.

## Conclusion

Almost three out of five CKD patients in Sub- Saharan African countries were suffering from anemia, which revealed that anemia is highly prevailing in patients with CKD. Its prevalence varies across the region with the highest prevalence in Cameroon. It was also found that stage of CKD, presence of DM, being female sex, and history of hemodialysis were significantly associated with the presence of anemia among CKD patients. Consequently, a high index of suspicion for anemia should be implemented among CKD patients particularly in patients with advanced CKD stage, history of hemodialysis, comorbid condition of DM, and female sex to improve their outcomes.

## Supporting information

S1 Table The guideline of Preferred Reporting Items for Systematic reviews and Meta-Analysis (PRISMA) checklist) (S1 Table). (DOCX)

S2 Table Methodological quality assessment of included studies using modified Newcastle—Ottawa Scale (NOS). (DOCX)

S3 Table The risk of bias assessment tool for the included studies. (DOCX)
