# Supplementary material for: Anemia and its predictors among chronic kidney disease patients in Sub-Saharan African countries: A systematic review and meta-analysis
Source: PLoS One. 2023 Feb 2;18(2):e0280817. doi: 10.1371/journal.pone.0280817 (PMC9894480; doi:10.1371/journal.pone.0280817)
Supplement: S2 Table — (DOCX) [file pone.0280817.s002.docx]

**Methodological quality assessment**

**S2 Table:** Methodological quality assessment of included studies using modified Newcastle - Ottawa Scale (NOS)

| **Corresponding author**  **[reference]** |  | **Criteria** | | | | | | | |  |
| --- | --- | --- | --- | --- | --- | --- | --- | --- | --- | --- |
|  |  | **Selection** | | | | **Comparability** | | **Outcome** | |  |
|  | **Study Design** | **Representativeness of the sample** | **Sample size** | **Non –responders** | **Ascertainment of exposure/ risk factor** | **The study controls for the most important factor** | **The study control for any additional factor** | **Assessment of the outcome** | **Statistical test** | **Quality score** |
| Adera et al.^31^ | Cross-sectional | B* | A* | A* | C | - | B* | A** | A* | 7 |
| Nalado et al.^22^ | Cross-sectional | B* | A* | C | C | A** | - | A** | A* | 7 |
| Alemu et al.^32^ | Cross-sectional | B* | A* | C | B* | A** | - | B** | A* | 8 |
| Meremo AJ et al.^34^ | Cross-sectional | A* | B | C | B* | A** |  | B** | A* | 7 |
| Akinola Oyekemi I et al.^26^ | Cross-sectional | B* | B | C | C | - | B* | A** | A* | 5 |
| Ijoma et al.^27^ | Cross-sectional | A* | B | C | B* | - | B* | A** | A* | 6 |
| C. K. MAINA et al.^36^ | Cross-sectional | B* | A* | C | C | - | B* | B** | A* | 6 |
| Raji, et al.^28^ | Cross-sectional | A* | A* | C | B* | A** | - | A** | A* | 8 |
| Abate et al.^16^ | Cross-sectional | A* | A* | C | B* | A** | - | A** | A* | 8 |
| George C, et al.^23^ | Retrospecive cohort | A* | B | C | B* | A** | - | A** | A* | 7 |
| Iyawe IO et al.^29^ | Cross-sectional | B* | B | C | C |  | B* | A** | A* | 5 |
| Emmanuel Oladipo Sanni, et al.^30^ | Cross-sectional | A* | B | C | B* | A** | - | A** | A* | 7 |
| Valerian Mwenda et al.^37^ | Cross-sectional | A* | A* | C | A** | A** | - | B** | A* | 9 |
| Bashir Abdrhman Bashir et al.^41^ | Cross-sectional | A* | B | A* | B* | A** | - | A** | A* | 8 |
| Temesgen Fiseha et al.^17^ | Cross-sectional | A* | A* | C | B* | A** | - | A** | A* | 8 |
| Aishatu Muhammad Nalado et al.^24^ | Cross-sectional | C | A* | C | C | A** | - | A** | A* | 6 |
| Francois Folefack Kaze et al.^38^ | cohort study | A* | A* | C | C | A** | - | A** | A* | 7 |
| Marie Patrice Halle et al.^39^ | Cross-sectional | C | A* | C | B* | - | B* | A** | B | 6 |
| L. HAUPT et al.^14^ | Cross-sectional | B* | B | C | C |  | B* | A** | A* | 5 |
| Namuyimbwa et al.^42^ | Cross-sectional | A* | A* | C | C | A** | - | A** | A* | 7 |
| Nalado et al.^25^ | Cross-section | A* | A* | C | B* | A** | - | A** | A* | 8 |
| Iyawe IO et al.^15^ | Cross-sectional | A* | A* | C | C | A** | - | A** | A* | 7 |
| Ruggajo P et al.^35^ | Cross-sectional | B* | A* | C | B* | A** | - | A** | A* | 8 |
| F. F. Kaze et al.^40^ | Cross-sectional | A* | B | C | B* |  | B* | A** | A* | 6 |
| Kidanewold A et al.^33^ | Cross-sectional | A* | A* | C | B* | A** |  | B** | A* | 8 |

*Note: from each item account point. (Accept the study for each study design based on total score of ≥50%)*

**Selection:** (Maximum 5 stars)
1) Representativeness of the sample: a) Truly representative of the average in the target population. * (all subjects or random sampling) .b) Somewhat representative of the average in the target population. * (nonrandom sampling) .c) Selected group of users.d) No description of the sampling strategy.
2) Sample size:a) Justified and satisfactory. *.b) Not justified.
3) Non-respondents: a) Comparability between respondents and non-respondents characteristics is
established, and the response rate is satisfactory. * .b) The response rate is unsatisfactory, or the comparability between respondents
and non-respondents is unsatisfactory. c) No description of the response rate or the characteristics of the responders and
the non-responders.
4) Ascertainment of the exposure (risk factor): a) validated measurement tool. ** .b) Non-validated measurement tool, but the tool is available or described.* c) No description of the measurement tool.
**Comparability:** (Maximum 2 stars)
1) The subjects in different outcome groups are comparable, based on the study design or analysis. Confounding factors are controlled. a) The study controls for the most important factor (select one). ** b) The study control for any additional factor. *
**Outcome:** (Maximum 3 stars)
1) Assessment of the outcome: a) Independent blind assessment. **,b) Record linkage. **,c) Self report. *,d) No description.
2) Statistical test:a) The statistical test used to analyze the data is clearly described and appropriate, and the measurement of the association is presented, including confidence intervals and the probability level (p value). *,b) The statistical test is not appropriate, not described or incomplete
