# Supplementary material for: Anemia and its predictors among chronic kidney disease patients in Sub-Saharan African countries: A systematic review and meta-analysis
Source: PLoS One. 2023 Feb 2;18(2):e0280817. doi: 10.1371/journal.pone.0280817 (PMC9894480; doi:10.1371/journal.pone.0280817)
Supplement: S3 Table — (DOCX) [file pone.0280817.s003.docx]

**Methodological quality assessment**

**S3 Table:** The risk of bias assessment tool for the included studies.

| **Corresponding author**  **[reference]** | Representation | Sampling | Random selection | Non-response bias | Data collected | Case definition | Reliability &validity of tool | Mode of data collection | Length prevalence period | Numerator & denominator | The overall risk of bias |
| --- | --- | --- | --- | --- | --- | --- | --- | --- | --- | --- | --- |
| Adera et al. [31] | Yes | Yes | Yes | No | Yes | Yes | Yes | Yes | Yes | Yes | Low risk |
| Nalado et al. [22] | Yes | Yes | Yes | Yes | Yes | Yes | Yes | Yes | Yes | Yes | Low risk |
| Alemu et al. [32] | Yes | Yes | Yes | No | Yes | Yes | Yes | Yes | Yes | Yes | Low risk |
| Meremo AJ et al. [34] | Yes | Yes | Yes | Yes | No | No | No | Yes | Yes | Yes | Moderate risk |
| Akinola Oyekemi I et al. [26] | Yes | Yes | Yes | Yes | No | Yes | No | Yes | Yes | Yes | Moderate risk |
| Ijoma et al. [27] | No | No | No | Yes | No | Yes | Yes | Yes | Yes | Yes | Moderate risk |
| C. K. MAINA et al. [36] | Yes | Yes | No | No | Yes | Yes | No | Yes | Yes | Yes | Moderate risk |
| Raji, et al. [28] | Yes | No | No | No | Yes | Yes | Yes | Yes | Yes | Yes | Moderate risk |
| Abate et al. [16] | Yes | Yes | Yes | Yes | Yes | Yes | Yes | Yes | Yes | Yes | Low risk |
| George C, et al. [23] | Yes | Yes | Yes | No | No | No | Yes | Yes | Yes | Yes | Moderate risk |
| Iyawe IO et al. [29] | Yes | Yes | No | No | Yes | Yes | No | Yes | Yes | Yes | Moderate risk |
| Emmanuel Oladipo et al. [30] | Yes | Yes | No | Yes | Yes | Yes | Yes | Yes | Yes | Yes | Moderate risk |
| Valerian Mwenda et al. [37] | Yes | Yes | Yes | Yes | Yes | No | No | No | Yes | Yes | Moderate risk |
| Bashir Abdrhman et al. [41] | No | No | No | Yes | Yes | Yes | Yes | Yes | Yes | Yes | Moderate risk |
| Temesgen Fiseha et al. [17] | Yes | Yes | Yes | Yes | Yes | Yes | Yes | Yes | Yes | Yes | Low risk |
| Aishatu Nalado et al. [24] | Yes | No | No | Yes | Yes | Yes | Yes | Yes | Yes | Yes | Moderate risk |
| Francois Kaze et al. [38] | No | No | No | Yes | No | Yes | Yes | Yes | Yes | Yes | Moderate risk |
| Marie Patrice Halle et al. [39] | Yes | No | No | No | Yes | Yes | Yes | Yes | Yes | Yes | Moderate risk |
| L. HAUPT et al. [14] | No | No | No | No | Yes | Yes | Yes | Yes | Yes | Yes | Moderate risk |
| Namuyimbwa et al. [42] | No | Yes | No | Yes | Yes | Yes | Yes | Yes | Yes | Yes | Moderate risk |
| Nalado et al. [25] | Yes | Yes | No | No | Yes | No | Yes | Yes | Yes | Yes | Moderate risk |
| Iyawe IO et al. [15] | Yes | No | Yes | No | Yes | Yes | Yes | Yes | No | Yes | Moderate risk |
| Ruggajo P et al. [35] | Yes | Yes | No | Yes | Yes | Yes | Yes | Yes | Yes | Yes | Low risk |
| F. F. Kaze et al. [40] | Yes | No | No | Yes | Yes | Yes | Yes | Yes | Yes | No | Moderate risk |
| Kidanewold A et al. [33] | Yes | Yes | Yes | No | Yes | Yes | Yes | Yes | Yes | Yes | Low risk |

- Note

Risk of bias assessment tool: Yes (low risk); No (high risk)

1. Representation: Was the study population a close representation of the target population?

2. Sampling: Was the sampling frame a true or close representation of the target population?

3. Random selection: Was some form of random selection used to select the sample OR was a census undertaken?

4. Non-response bias: Was the likelihood of non-response bias minimal?

5. Data collection: Were data collected directly from the subjects?

6. Case definition: Was an acceptable case definition used in the study?

7. Reliability and validity of study tool: Was the study instrument that measured the parameter of interest show to have reliability and validity?

8. Data collection: Was the same mode of data collection used for all subjects?

9. Prevalence period: Was the length of the prevalence period for the parameter of interest appropriate?

10. Numerators and denominators: Were the numerator(s) and denominator(s) for the parameter of interest appropriate?

The overall risk of bias scored based on the number of high risk of bias per study: low risk (>8), moderate risk (6–8), and high risk (≤5)
